# Impaired cardiac function in chronic fatigue syndrome measured using magnetic resonance cardiac tagging

**DOI:** 10.1111/j.1365-2796.2011.02429.x

**Published:** 2011-08-15

**Authors:** K G Hollingsworth, T Hodgson, G A MacGowan, A M Blamire, J L Newton

**Affiliations:** 1 Newcastle Magnetic Resonance Centre, Institute of Cellular Medicine, Newcastle UniversityNE4 5PL; 2 Department of Cardiology, Freeman Hospital, Newcastle upon Tyne, NE7 7DN and Institute of Genetic Medicine, Newcastle UniversityNE1 3BZ; 3 Institute for Ageing and Health, Newcastle UniversityNE4 5PL; Newcastle upon Tyne, UK

**Keywords:** cardiac, CFS, fatigue, MRI, tagging, torsion

## Abstract

**Objectives:**

Impaired cardiac function has been confirmed in patients with chronic fatigue syndrome (CFS). Magnetic resonance cardiac tagging is a novel technique that assesses myocardial wall function *in vivo*. We hypothesized that patients with CFS may have impaired development and release of myocardial torsion and strain.

**Methods:**

Cardiac morphology and function were assessed using magnetic resonance imaging and cardiac tagging methodology in 12 CFS patients (Fukuda) and 10 matched controls.

**Results:**

Compared to controls, the CFS group had substantially reduced left ventricular mass (reduced by 23%), end-diastolic volume (30%), stroke volume (29%) and cardiac output (25%). Residual torsion at 150% of the end-systolic time was found to be significantly higher in the patients with CFS (5.3 ± 1.6°) compared to the control group (1.7 ± 0.7°, *P* = 0.0001). End-diastolic volume index correlated negatively with both torsion-to-endocardial-strain ratio (TSR) (*r* = −0.65, *P* = 0.02) and the residual torsion at 150% end-systolic time (*r* = −0.76, *P* = 0.004), so decreased end-diastolic volume is associated with raised TSR and torsion persisting longer into diastole. Reduced end-diastolic volume index also correlated significantly with increased radial thickening (*r* = −0.65, *P* = 0.03) and impaired diastolic function represented by the ratio of early to late ventricular filling velocity (E/A ratio, *r* = 0.71, *P* = 0.009) and early filling percentage (*r* = 0.73, *P* = 0.008).

**Conclusion:**

Patients with CFS have markedly reduced cardiac mass and blood pool volumes, particularly end-diastolic volume: this results in significant impairments in stroke volume and cardiac output compared to controls. The CFS group appeared to have a delay in the release of torsion.

## Introduction

Recent studies using a range of assessment modalities have shown that chronic fatigue syndrome (CFS) is associated with abnormalities of cardiac function [1–3]. Echocardiographic and impedance studies have confirmed impaired cardiac contractility [2], reduced left ventricular (LV) function, end-diastolic dimensions and cardiac output [1, 1] with magnetic resonance spectroscopy detecting impaired cardiac bioenergetic function [2]. The severity of these cardiac abnormalities also appears to relate to symptom severity [2, 2]. This has lead to the suggestion that those with CFS have a primary cardiac abnormality that accounts for at least some of their symptoms.

**Fig. 1:**
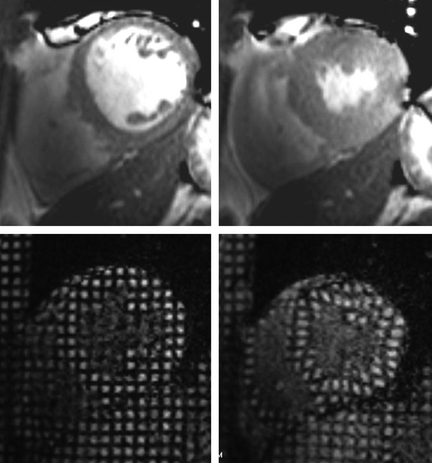
Cardiac cine imaging (top) and cardiac tagging (bottom) at diastole (left) and systole (right), showing how a rectangular grid of nulled signal applied at diastole remains with the tissue through the cardiac cycle, allowing calculation of strain and torsion.

**Fig. 2:**
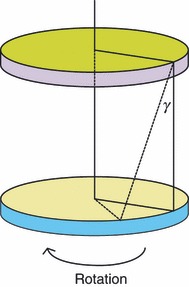
Tagging two parallel short-axis slices allows the calculation of the torsion (the longitudinal-circumferential shear angle, γ) between the two planes as shown.

Whilst standard cine magnetic resonance imaging (MRI) provides the gold standard measures of cardiac morphology and function, it cannot give detailed information about myocardial transmural strain (percentage shortening) and torsion (a measure of ‘twist’), which are affected by energetic deficits before becoming apparent as a clinical impairment. The torsion developed in systole arises from the relative effects of fibre contraction across the cardiac wall from epicardium to endocardium [5], and the release of torsion (‘untwisting’) is an index of diastolic function which is independent of volume change. Given the observations of functional abnormalities of cardiac function in CFS [1–4], we hypothesized that we would be able to detect impairments in the development and release of cardiac strain and torsion in patients with CFS compared to an age-matched control group. Such measurements can be taken using cardiac MRI tagging [6]. This method works by nulling signal from the myocardium in diastole in a rectangular grid pattern and tracking the deformation of these tags through the rest of the cardiac cycle (Fig. 1). By tagging two parallel planes, it is possible to calculate myocardial torsion (Fig. 2), and in-plane analysis allows circumferential strains to be calculated across the myocardial wall. The technique has been used to examine cardiac function during healthy ageing [7–9], where gradual subclinical differences in systolic and diastolic function are expected, as well as in conditions with definite cardiac involvement [10, 11].

Using this approach, we studied a well-characterized group of patients with CFS, with reference to matched controls, to determine whether there were quantifiable abnormalities of cardiac strain or torsion.

## Subjects and methods

### Subjects

Twelve female subjects with CFS were identified via the UK patient support group ‘ME North East’. Subjects had been diagnosed with CFS in a specialist CFS service within 2 years of assessment and all fulfilled the Fukuda diagnostic criteria [12, 12]. Ten age-, weight- and height-matched sedentary female subjects were recruited as controls: all controls performed less than 30 min exercise three times per week (Table 1). A minimum group size of 10 was determined to have sufficient power to detect a 5% change in LV mass between controls and patients, based on previous control data. Both patients and controls were excluded if taking any medication that could influence the assessment of haemodynamics (e.g. beta blockers, calcium antagonists and antidepressants), whether diabetic or with renal or hepatic disease. Subjects were excluded if they are not in sinus rhythm, are unable to stand, are unable to attend for assessment or had prior evidence of cardiac disease. Controls were screened to exclude subjects with hypertension (systolic blood pressure > 150 mmHg) or abnormalities on 12-lead ECG. Details are shown in Table 1. Written informed consent was obtained from all participants, and institutional ethical approval was obtained.

**Table 1:** Cardiac parameters for controls and chronic fatigue syndrome (CFS) subjects. Student t-test or Mann–Whitney U test used for comparison with Bonferroni correction for multiple comparisons

	Control	CFS	*P*-value
Age (year)	51 ± 8	50 ± 12	ns
BMI (kg m^−2^)	27 ± 3	24 ± 4	ns
Weight (kg)	71 ± 7	64 ± 10	ns
Systolic blood pressure (mmHg)	121 ± 13	128 ± 27	ns
Diastolic blood pressure (mmHg)	77 ± 11	81 ± 14	ns
LV mass (g)	96 ± 16	74 ± 11	0.028
LV index (g m^−2^)	54 ± 7	44 ± 6	ns
Ejection fraction (%)	62 ± 7	64 ± 9	ns
LV mass/End-diastolic volume (g mL^−1^)	0.78 ± 0.07	0.87 ± 0.18	ns
Stroke volume (mL)	76 ± 13	54 ± 7	0.0015
Stroke index (mL m^−2^)	43 ± 7	32 ± 4	0.007
Heart rate (bpm)	64 ± 12	67 ± 9	ns
Cardiac output (L min^−1^)	4.8 ± 0.6	3.6 ± 0.7	0.01
Cardiac index (mL m^−2^)	2.8 ± 0.4	2.2 ± 0.4	0.04
End-diastolic volume (mL)	123 ± 21	86 ± 14	0.0027
End-diastolic volume index (mL m^−2^)	69 ± 11	52 ± 8	0.014
End-systolic volume (mL)	47 ± 14	32 ± 11	ns
End-systolic volume index (mL m^−2^)	26 ± 7	19 ± 6	ns
E/A ratio (−)	1.7 ± 0.6	1.8 ± 1.0	ns
Early filling percentage (%)	72 ± 5	70 ± 9	ns
Torsion-to-endocardial-strain ratio	0.46 ± 0.11	0.54 ± 0.15	ns
Peak torsion (degrees)	6.2 ± 1.7	7.7 ± 1.9	ns
Residual torsion at 150% ES (degrees)	1.7 ± 0.7	5.3 ± 1.6	0.0001
Peak circumferential strain (%)	18.3 ± 1.7	18.7 ± 2.1	ns
Longitudinal shortening (%)	18.3 ± 3.1	19.3 ± 2.2	ns
Radial thickening (%)	61.3 ± 17.2	83.5 ± 30.4	ns

### Cardiac magnetic resonance cine imaging

Cardiac examinations were performed using a 3T Philips Intera Achieva scanner (Best, the Netherlands). A dedicated 6-channel cardiac coil (Philips) was used with the subjects in a supine position and electrocardiogram (ECG) gating (Philips vectorcardiogram, VCG system). Cardiac magnetic resonance cine imaging was acquired to assess cardiac morphology, systolic and diastolic function. A stack of balanced steady-state free precession images was obtained in the short-axis view during breath holding covering the entire left ventricle (FOV = 350 mm, TR/TE = 3.7/1.9 ms, turbo factor 17, flip angle 40°, slice thickness 8 mm, 0 mm gap, 14 slices, 25 phases, resolution 1.37 mm, temporal duration approximately 40 ms per phase dependent on heart rate): perpendicular long-axis views were also acquired. Image analysis was performed using the cardiac analysis package of the ViewForum workstation (Philips). Manual tracing of the epicardial and endocardial borders was performed on the short-axis slices at end systole and end diastole. The algorithm for contour selection and subsequently calculating left ventricular mass, systolic and diastolic parameters, including the ratio of early to late ventricular filling velocity (E/A ratio) and early filling percentage, have been detailed elsewhere [13]. The ratio of LV mass to the end-diastolic volume is reported as this is often quoted as an index of concentric hypertrophy in ageing studies [14].

### Cardiac tagging

Tagged images of the myocardium in the short axis were obtained at the same session as the morphological imaging using the same cardiac coil (Fig. 1). A multishot turbo-field echo sequence with TFE factor 9 was used (TR/TE/FA/NEX = 4.9/3.1/10°/1, SENSE factor 2, FOV 350 × 350mm, voxel size 1.37 × 1.37 mm with an orthogonal CSPAMM grid with tag spacing of 7 mm [15]. Two adjacent short-axis slices of 10 mm thickness were acquired at mid-ventricle with a 2-mm gap. The Cardiac Image Modelling package (Auckland UniServices Ltd, Auckland, New Zealand) was used to analyse the tagging data by aligning a mesh on the tags between the endo- and epicardial contours. Circumferential strain and the rotation of the two planes were calculated throughout the cardiac cycle. The torsion between the two planes (taken as the circumferential-longitudinal shear angle,γ, Fig. 2) was calculated as previously described [8] to account for the radius of the ventricle. In the healthy heart, torsion occurs such that there is homogeneity of fibre shortening across the myocardial wall and is a marker of the dominance of epicardial fibres over endocardial fibres as a consequence of the greater radius in the epicardium. To quantify the relationship between torsion and strain, we calculated a torsion-to-endocardial-strain ratio (TSR) [6, 6] as the ratio of the peak torsion (in radians), defined as the shear angle between two planes on the epicardial surface [6], and the peak circumferential strain in the endocardial third of the myocardium [6, 6]. This ratio has been shown to be near constant amongst healthy subjects and to increase with both healthy ageing and disease [6, 6].

The rate at which torsion dissipates after systole is a further important measure, and this was assessed by calculating the residual torsion at 150% of the end-systolic time [7], end systole being defined from the cine imaging. Where this falls between two tagging acquisition times, linear interpolation of the nearest results was used. Longitudinal shortening was determined from cine MRI in the 4-chamber view by determining the perpendicular distance from the plane of the mitral valve to the apex in systole and diastole and expressing the difference in the measures as a percentage of the diastolic value. The myocardial wall thickness at systole and diastole was determined from the standard imaging at the same mid-ventricular level as the cardiac tagging by averaging the distance between the epicardial and endocardial countours around the left ventricle, and the percentage increase in wall thickness (radial thickening) from diastole to systole was calculated.

### Image and statistical analysis

Image analysis was performed blinded to the status of patients and controls. Statistical comparisons were made using SPSS version 17 (IBM, Armonk, NY, USA). Data were tested for normality using the Shaprio–Wilk test: only systolic blood pressure, cardiac-mass-to-volume ratio, E/A ratio and radial thickening were found to be nonparametric. Parametric variables are presented as mean and standard deviation and comparisons were made between groups using Student’s *t*-test with Bonferroni correction for multiple comparisons. Comparisons for nonparametric variables were made using the Mann–Whitney *U* test with correction for multiple comparisons. Correlations were executed as a two-tailed test using the Pearson correlation method. Statistical significance level was set at *P* < 0.05.

## Results

### Cardiac morphology and function by cine MRI

Table 1 summarizes the morphological and functional parameters. The patients with CFS had substantially reduced LV mass (by 23% compared to control), end-diastolic volume (30%), stroke volume (29%) and cardiac output (25%) compared to the controls. When normalized to the body surface area to eliminate the effect of individual body size, stroke index (26%), cardiac index (21%) and end-diastolic volume index (25%) were all significantly reduced.

There were no significant differences in resting heart rate or in left ventricular ejection fraction between patients with CFS and matched controls. The ratio of LV mass to end-diastolic volume did not differ significantly between the patients with CFS and controls, though some CFS individuals had a high ratio, influenced principally by low end-diastolic volume.

### Cardiac tagging measurements

**Fig. 3:**
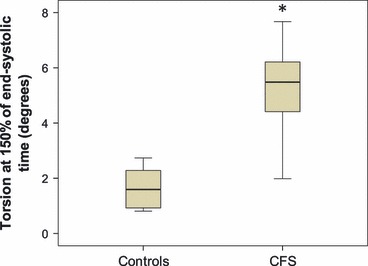
Residual torsion at 150% of end-systolic time in control and patients with PBC.*P < 0.0001.

Neither TSR nor peak torsion was significantly raised in the CFS group as a whole (Table 1), though some individuals had peak torsion and TSR greater than the control group. However, the residual torsion at 150% of the end-systolic time was found to be significantly higher in the patients with CFS (5.3 ± 1.6°) compared to the control group (1.7 ± 0.7°, Fig. 3). Increased residual torsion at 150% of end-systolic time correlated significantly with reduced diastolic function represented by the E/A ratio (*r* = −0.79, *P* = 0.002). Longitudinal shortening and radial thickening were not found to be different between patients and controls, though some CFS individuals had high radial thickening.

### Relationship between cardiac tagging parameters and end-diastolic volume changes in CFS

Within the CFS group, end-diastolic volume index correlated negatively with both TSR (*r* = −0.65, *P* = 0.02) and the residual torsion at 150% end-systolic time (*r* = −0.76, *P* = 0.004), so decreased end-diastolic volume was associated with raised TSR and torsion persisting longer into diastole. Reduced end-diastolic volume index also correlated significantly with increased radial thickening (*r* = −0.65, *P* = 0.03), impaired diastolic function represented by E/A ratio (*r* = 0.71, *P* = 0.009) and early filling percentage (*r* = 0.73, *P* = 0.008).

## Discussion

This study examined cardiac morphology and function in patients with CFS using advanced MRI cardiac imaging and tagging techniques for the first time. Patients with CFS were found to have markedly reduced cardiac mass and blood pool volumes, particularly end-diastolic volume: this results in significant impairments in stroke volume and cardiac output compared to controls. Whilst peak cardiac strain and torsion were not found to be groupwise different from controls, the residual torsion at 150% of the end-systolic time was much greater than controls, indicating a delay in the release of torsion. Despite no overall groupwise difference, some individuals had increased TSR and radial thickening: such changes were found to be associated with reduced end-diastolic volume.

Reduction in end-diastolic volume (preload) is strongly suggestive of a marked reduction in the total blood volume in patients with CFS which is in keeping with other studies [16]. Previous measurements using ultrasound [3, 17] had previously identified reduced cardiac output in severe patients with CFS (defined by a list of symptoms), but the magnitude of the reductions found in that cohort was much less than those found here. In one study [3], it was possible to show that the difference in cardiac output and end-diastolic volume could be entirely accounted for by reduction in the total blood volume. Such reductions in CFS have been termed ‘small heart syndrome’. However, these studies did not measure the marked changes in LV mass between patients with CFS and controls found here, and it is not clear that there is such a simple explanation for this difference. This cross-sectional study cannot establish whether reduced LV mass results from prolonged reduction in blood pool size and low end-diastolic volume and this warrants further longitudinal study.

Analysis of cardiac deformation by tagging demonstrated that the reduction in end-diastolic volume index was associated with increased TSR and in increased radial thickening of the myocardial wall between diastole and systole in some individuals. These changes could occur as a geometric consequence of cardiac contraction to a smaller radius caused by low blood volume: the greater difference in circumferential strain between the endo- and epicardial walls at smaller radii will be balanced by greater torsion [5], and increased contraction of the endocardial wall will lead to enhanced radial thickening [18, 19]. Alternatively, the magnitude of reduced end-diastolic volume and increased TSR may be correlated because they are both sequelae of the CFS disease process, with increased TSR representing impaired subendocardial function in some individuals [5].

This pattern of increased cardiac TSR associated with decreased blood pool volume stands in contrast to our previous work [9] on healthy subjects in young (mean age 31 ± 6 years), middle-aged (mean age 50 ± 9 years) and older groups (mean age 62 ± 2 years), where we find that the TSR remains constant between the young and middle-aged groups (mean 0.45 and 0.46 respectively), whilst it is found to be raised by 41% in the oldest group (mean 0.62). Other authors have noted increases in torsion and TSR, notably [6], where a 38% increase in TSR and 33% increase in torsion were noted between two groups of healthy volunteers with mean ages of 23 and 68 years. In these groups, there were no substantial changes in end-diastolic volume which associate with changes in TSR.

The timely release of torsion and strain during diastole is crucial for good diastolic function, and we evaluated this, as in other studies [7], by measuring the residual torsion at 150% of the end-systolic time. The CFS group had 200% more residual torsion than controls. By contrast, our work on healthy controls [9] indicates a 38% increase in residual torsion between mean ages of 50 and 62 years. Similarly, a study with a wider age range [7] found a 56% increase in residual torsion between the ages of 22 and 69 years. This impairment in torsion as a result of ageing may be due to the inability to resequester Ca^2+^ ions into the sarcoplasmic reticulum sufficiently quickly after systole to permit cardiac relaxation: whilst there is no established theory as to why this would occur in CFS, the consequences of hypovolaemia on Ca^2+^ resequestration are presently unclear. Indeed, the increased residual torsion at 150% of end-systolic time in CFS does correlate with lower early filling in diastole, demonstrating the anticipated diastolic consequences of delay in the release of torsion.

The limitations of this study are the small number of individuals studied its cross-sectional nature which means that we are unable to chart the time course with which cardiac impairments occur. Correlation tests were not corrected for multiple comparisons, though groupwise difference tests were. As this was part of a larger MR study, only two slices of cardiac tagging data were acquired at mid-ventricle. However, by matching a control population for gender, age, blood pressure and weight, we have been able to demonstrate changes in cardiac parameters that are more sensitive than a comparison against global reference ranges for the entire population. The patients with CFS in this study were recruited through a patient support group potentially introducing recruitment bias versus the general CFS population, though they all had recent diagnosis of CFS according to established criteria.

Potentially, these findings suggest that therapies that can correct the low cardiac blood volume may be helpful: this might include graded exercise therapies, which might improve cardiac blood volume [20]. Anecdotally patients (http://www.davidsbell.com/LynNewsV3N2.htm) describe symptomatic improvements with the administration of intravenous fluid. Our findings would point towards a possible explanation for this subjective improvement, and future work will include interventions to restore fluid volume in patients with CFS and explore the potential amelioration of the cardiac functional impairments seen in the present study, including the progressive normalization of LV mass. Such a study would establish the primacy of blood volume reduction and determine whether there are primary myocardial deficits that are not associated with low blood volume.
